# Thermoluminescence-Based Detection of Irradiation in Valerian Root (*Valeriana officinalis*) Dietary Supplements: A Pilot Study on Undeclared Treatment and Labelling Compliance

**DOI:** 10.3390/molecules31173016

**Published:** 2026-08-28

**Authors:** Jarosław Chmielewski, Michał Sroka, Barbara Gworek, Ewa Górska, Piotr Szenk, Edyta Hewelke, Monika Kaczoruk

**Affiliations:** 1 Faculty of Health Sciences, Cardinal Stefan Wyszyński University, 01-938 Warsaw, Poland; j.chmielewski@uksw.edu.pl; 2 Independent Researcher, London W1W 5PF, UK; msroka14138@kampusamh.pl; 3 Department of Environmental Chemistry and Risk Assessment, The Institute of Environmental Protection—National Research Institute, 02-170 Warsaw, Poland; barbara.gworek@ios.edu.pl; 4 Department of Biochemistry and Microbiology, Institute of Biology, Warsaw University of Life Sciences, 02-776 Warsaw, Poland; ewa_gorska@sggw.edu.pl; 5 Faculty of Medicine, Lazarski University, 02-662 Warsaw, Poland; piotr.szenk@gmail.com; 6 Department of Environmental Development and Remote Sensing, Warsaw University of Life Sciences, 02-787 Warsaw, Poland; edyta_hewelke@sggw.edu.pl; 7 Institute of Rural Health, 20-090 Lublin, Poland

**Keywords:** dietary supplements, valerian, *Valeriana officinalis*, food irradiation, ionizing radiation, thermoluminescence, technological authenticity, food labelling, market surveillance, food fraud

## Abstract

Background/Objectives: Food irradiation is a recognized method for the microbiological decontamination and preservation of plant raw materials; however, in the European Union, its use is subject to strict legal regulation and to a mandatory labelling obligation. For dietary supplements, which are legally classified as foodstuffs, this issue is particularly important for market transparency and the consumer’s right to information. The aim of this pilot study was to assess the presence of post-irradiation characteristics in selected dietary supplements available at retail in Poland, with particular attention to products containing valerian root (*Valeriana officinalis* L.), and to compare the results obtained with the information declared on the product labels. Methods: The analysis included 15 analytical samples representing 11 commercial formulations. The study was performed using the thermoluminescence method according to standard PN-EN 1788:2002 in an ISO/IEC 17025-accredited laboratory. Results: Thermoluminescence characteristics consistent with prior irradiation were observed in 7 samples, whereas 8 samples did not show such properties. Positive results concerned exclusively products containing valerian root, including replicate batches of two commercial formulations. None of the positive samples carried any label information about irradiation or exposure to ionizing radiation. The results indicate that some dietary supplements on the market may be subjected to irradiation without this being properly reflected in their labelling. Conclusions: Given the pilot nature of the study and the limited, non-random sample, the results should be treated as a premise for further analysis rather than as a basis for generalizing to the entire market. The results of this study indicate a need for broader analytical monitoring and targeted control of dietary supplements containing plant raw materials.

## 1. Introduction

Dietary supplements containing plant components are among the fastest-growing categories of food products. Their popularity may be associated, among other factors, with a broader wellness orientation, reflecting a growing focus of individuals on health promotion, disease prevention, and the use of natural-origin products and preparations that support psychophysical well-being [1,2,3]. Supplements based on herbal raw materials with a long history of use—such as valerian root (*Valeriana officinalis* L.), commonly used in preparations that support sleep and help to calm and reduce nervous tension—hold a particular position within this group. At the same time, these products are especially sensitive from the perspective of technological transparency, because consumers usually purchase them assuming that they are dealing with minimally processed, natural raw materials that remain relatively close to their original form [1,2,3].

In technological practice, however, the production of plant-based dietary supplements is considerably more complex. Plant raw materials—especially root materials—are exposed to microbiological contamination through contact with soil and during harvesting, drying, grinding, storage, and transport [4,5,6,7]. For this reason, producers and raw-material suppliers may need to apply decontamination treatments to meet quality and microbiological safety requirements. One of the methods used for this purpose is food irradiation, i.e., exposing a product or its ingredients to a controlled dose of ionizing radiation [4,5,6,7,8].

Food irradiation is a non-thermal technology that may be carried out using gamma radiation, X-rays, or accelerated electrons [4,5,6,7,8]. Its main relevant effect in this regard is the inactivation of pathogenic and saprophytic microorganisms through direct and indirect damage to nucleic acids, membranes, and other cellular structures [4,5,6,7,8]. Numerous international assessments indicate that, provided appropriate technological conditions are maintained, irradiation can be an effective and safe method of food preservation [8,9,10,11]. From the perspective of the present study, however, what is crucial is not the safety of the technology itself, but the obligation to disclose its use to the consumer.

In the European Union, these issues are regulated by Directives 1999/2/EC and 1999/3/EC, as well as by Regulation (EU) No 1169/2011 on the provision of food information to consumers [12,13,14]. These guidelines specify the conditions for the use of ionizing radiation in food processing, the rules for authorizing specific categories of food for such treatment, and the labelling requirements [12,13]. At the EU level, the harmonized list covers dried aromatic herbs, spices, and vegetable seasonings [13]. In Poland, the relevant provisions were implemented through, among others, the Act on Food and Nutrition Safety and the Regulation of the Minister of Health of 20 June 2007 on the irradiation of food with ionizing radiation [15,16]. Dietary supplements are legally classified as foodstuffs; therefore, the requirements concerning consumer information about the exposure of a product or its component to ionizing radiation also apply to them [12,13,14,15,16].

It should be emphasized that irradiated food does not become radioactive. Irradiation does not involve introducing radioactive isotopes into the product; rather, it delivers radiation energy that can induce changes in the biological and inorganic materials present in the product [8,17]. This means that detecting prior irradiation requires methods that identify permanent physicochemical markers of exposure rather than measuring the product’s radioactivity. One such method is thermoluminescence (TL), which is especially useful for materials containing silicate minerals [18,19,20,21].

The physicochemical basis of thermoluminescence is well established. Minerals such as quartz and feldspar, present as natural impurities in plant materials, possess crystal-lattice defects that can act as electron traps. Under ionizing radiation, some electrons are excited and become trapped at these defects. During subsequent controlled heating of the material, the trapped electrons are released, return to the ground state, and emit light. The intensity and temperature profile of this emission depend on the energy depth of the traps and on the radiation history of the material [19,20,21]. Analysis of the resulting glow curves can therefore provide reliable information on prior irradiation.

In regulatory assessment practice, what matters is not only the fact that ionizing radiation was used, but also the transparency of its declaration. When a product shows signs of prior irradiation but is not appropriately labelled, a discrepancy arises between the product’s technological history and the information provided to the consumer. In this framing, the problem can be regarded not only as a matter of labelling compliance, but also as a question of the product’s technological authenticity—and potentially as a form of technological food adulteration, i.e., a type of food fraud.

Data on the undeclared irradiation of dietary supplements are limited, especially for plant products sold at retail in Poland and other Central and Eastern European countries [20,22,23]. For products containing valerian root, this gap is particularly important, because this raw material may combine two factors that increase the likelihood of signal detection: an elevated microbial load characteristic of root-based materials, and the presence of a mineral fraction enabling effective TL analysis [4,5,6,7,19,20].

## 2. Results

### 2.1. Characteristics of the Studied Product Pool

A total of 15 analytical samples were analyzed, representing 11 commercial formulations. The pool included capsules and tablets containing valerian root as the sole active ingredient, composite products containing valerian root and black pepper, multi-component preparations containing valerian together with other herbal components, and comparator products without valerian (e.g., bacopa, turmeric, garcinia, and vitamin supplements). In no case did the product label carry a declaration of irradiation or exposure to ionizing radiation.

### 2.2. Results of Thermoluminescence Analysis

Characteristics of prior irradiation were observed in 7 of the 15 analytical samples (46.7%). All positive samples contained valerian root, either alone or in combination with black pepper. Positive results were obtained for samples A, E1, E2, E3, K1, K2, and K3. The remaining 8 samples (B, C, D, F, G, H, I, J) were classified as negative. In the positive samples, the natural TL1 signal was clear from the first reading and showed a broad maximum in the high-temperature region. In the negative samples, the natural signal was negligible or limited to background levels, and a clear luminescence signal appeared only after the calibration dose.

#### 2.2.1. Relationship Between Raw-Material Type and Result

All positive results concerned products containing valerian root, indicating a strong association between plant-matrix type and a positive TL profile. At the same time, not all valerian-containing products were positive: several multi-ingredient preparations containing valerian remained negative (F, H, I, J). The mere declared presence of *Valeriana officinalis* is therefore an insufficient explanation of the phenomenon. The form of the raw material, its origin and supplier, its degree of fragmentation, and technological practices specific to a given production line may all be relevant.

#### 2.2.2. Inter-Batch Repeatability

A particularly strong interpretive argument was the consistency of results within batches of two products. All three analyzed batches of product K (K1–K3) were positive, and all three analyzed batches of product E (E1–E3) were positive. This full repeatability across independent batches argues against an accidental analytical artifact or incidental contamination of a single batch.

#### 2.2.3. Statistical Comparison

Among the simple valerian preparations (valerian root alone or with black pepper), the proportion of positive results was 100% (7/7), whereas among the remaining supplements it was 0% (0/8). Fisher’s exact test indicated a statistically significant difference between the groups (*p* ≈ 0.0002; *p* = 1.55 × 10^−4^), which is inconsistent with accidental irradiation and points to a systematic application of this treatment to the *Valeriana officinalis* raw material used in these preparations.

### 2.3. Label Audit

**Figure 1:**
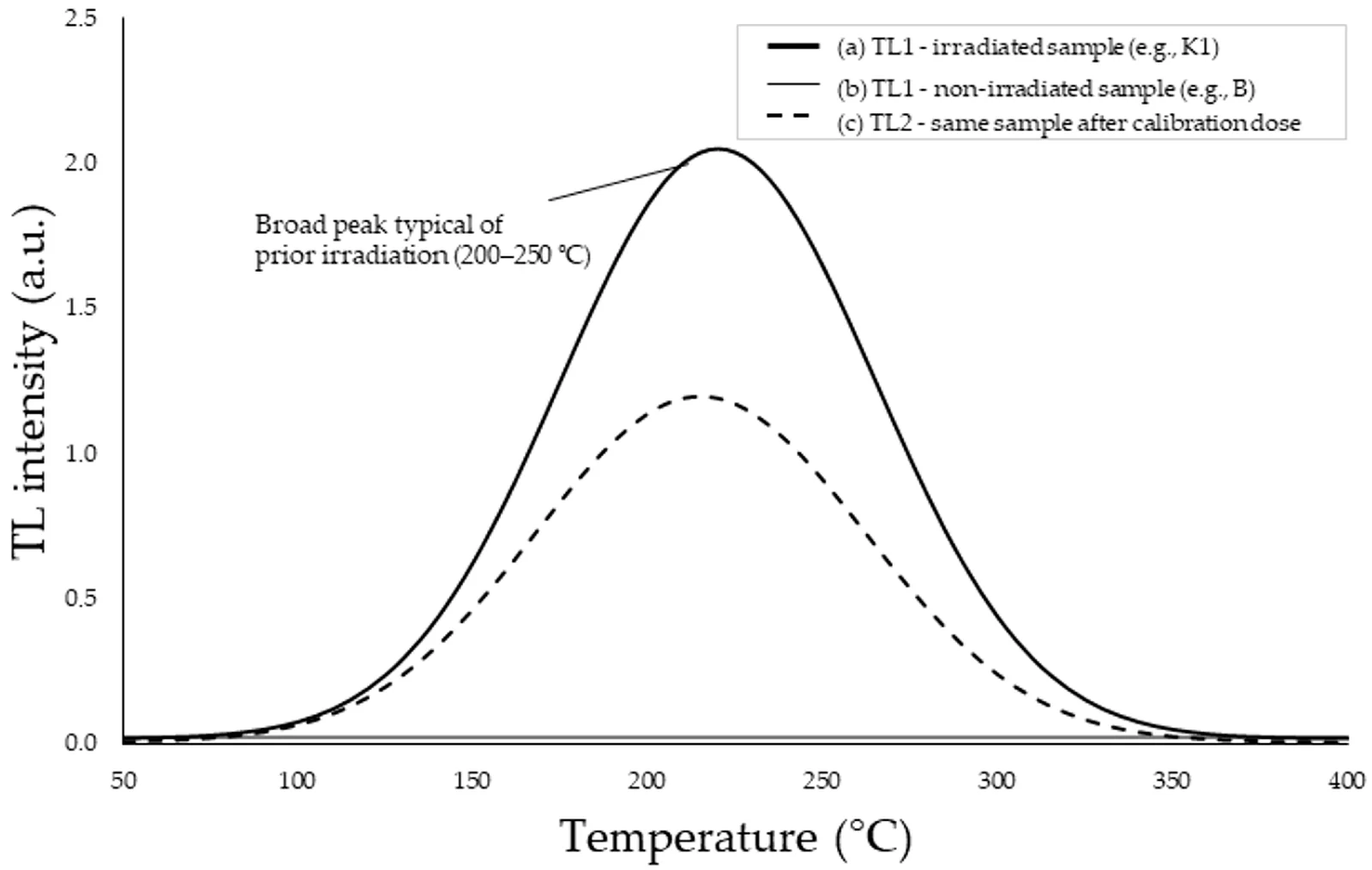
Representative thermoluminescence glow curves. Curve (a): natural TL1 signal of a positive (irradiated) sample, with a broad emission maximum at ~200–250 °C, characteristic of a material previously exposed to ionizing radiation. Curve (b): natural TL1 signal of a non-irradiated sample, at background level. Curve (c): the same sample as in (b) after the TL2 calibration dose, confirming the presence of a mineral fraction capable of thermoluminescence.

In all 15 cases, no information about irradiation appeared on the label. Consequently, for the 7 positive samples a discrepancy was found between the analytical result and the product labelling: the laboratory-detected signs of prior irradiation were not reflected in the information provided to the consumer. Detailed results are presented in Table 1 and Table 2, and in Figure 1.

## 3. Discussion

The results show that some of the analyzed dietary supplements available at retail in Poland exhibit characteristics of prior irradiation, and that this phenomenon is limited to selected products containing valerian root. None of the positive products carried a declaration concerning irradiation or exposure to ionizing radiation. From a scientific and regulatory standpoint, this reveals a discrepancy between the product’s technological history and the information provided to consumers.

What matters most is not the overall percentage of positive results, but their pattern. Positive signals were confined to selected valerian preparations and were repeatable across independent batches of two formulations. At the same time, not all valerian-containing products were positive. This argues against interpreting a positive result as a simple function of the presence of a given plant species. More plausibly, the observed effect reflects a combination of factors, including the origin of the raw material, the type and degree of its fragmentation, the mineral-fraction content, the microbiological load, and the technological practices applied by specific suppliers or manufacturers.

From a physicochemical standpoint, the interpretation of a positive result rested not on signal detection alone, but on the qualitative assessment of the glow-curve profile. In the positive samples, a broad, stable emission maximum in the high-temperature region was observed, consistent with electrons trapped at deeper energy levels of the mineral lattice. This signal is more durable and less prone to fading than low-temperature signals, and its presence strengthens the interpretation of prior irradiation [19,20,21]. In the negative samples, a low natural signal with a clear response only after the calibration dose confirms that TL-capable mineral material was present but showed no trace of prior irradiation.

The results can also be considered from a biological and technological perspective. Root materials such as valerian root are more exposed to soil contact and microbiological contamination than many other plant materials [4,5,6,7] and may therefore require more frequent decontamination, particularly for supplements intended for long-term storage. In this sense, the positive results for some valerian preparations are biologically and technologically plausible. The present study cannot determine whether irradiation was applied to the raw material, an intermediate, or the final product.

The problem revealed here should not be equated with a statement that the examined products are harmful to health. When applied in accordance with regulations and good practices, food irradiation is a recognized preservation method [8,9,10,11,17]. The key issue is the transparency and compliance of the declaration. When a product shows signs of prior irradiation and the label lacks the required information, a discrepancy arises that can be considered in terms of the product’s technological authenticity, and potentially as technological food adulteration—a form of food fraud. Nonetheless, caution is warranted: the present study does not establish intent, does not identify the supply-chain stage at which irradiation occurred, and does not allow responsibility to be assigned to any specific party.

From a practical standpoint, the study confirms the usefulness of thermoluminescence analysis for identifying prior irradiation in products containing a mineral fraction [19,20,21]. The findings also have implications for supervision of the dietary-supplement market: these products are widely available in pharmacies, online, and at retail, and routine inspections do not always include a detailed assessment of their technological history. Independent laboratory analyses may therefore provide valuable information complementary to standard control procedures [24,25,26,27].

This study has important limitations. First, the small, purposeful sample precludes generalization to the entire market. Second, the TL method was applied here for identification rather than dosimetry, so it detects characteristics of prior irradiation but does not allow full dose reconstruction. Third, data from manufacturers and suppliers were not available, which would have enabled a more complete interpretation of the origin of the positive results. Finally, the phytochemical composition of the examined products was not analyzed in parallel. Assessing the effect of irradiation on the phytochemical profile of valerian root (for example, the stability of valerenic acids) lies beyond the scope of the present identification study and is proposed only as a direction for future work.

## 4. Materials and Methods

The aim of the study was to assess the presence of characteristics of prior irradiation in selected dietary supplements available at retail in Poland, using the thermoluminescence method according to standard PN-EN 1788:2002 [28]. It was hypothesized that some retail dietary supplements containing plant raw materials may exhibit properties of prior irradiation despite the absence of appropriate labelling, and that this may more frequently concern preparations containing dried root materials—especially valerian root—owing to a higher risk of microbiological burden and the associated need for decontamination [4,5,6,7].

The study was designed as a pilot, exploratory laboratory analysis of products present in legal trade in Poland. The research material comprised 15 analytical samples representing 11 commercial dietary-supplement formulations. Products were purchased from pharmacies, retail outlets, and the online stores of manufacturers and distributors. Sample selection was purposeful and not randomized; the design was intended to identify a potential signal of the problem within the specified category of plant products, not to estimate the frequency of exposure across the entire dietary-supplement segment.

GenAI tools were only used to help prepare the graphical abstract. No GenAI tools were used to generate, analyse, interpret or modify the study data, nor to draft the manuscript, draw scientific conclusions or decide on the content of the manuscript. The authors reviewed and approved the final graphical abstract, and accept full responsibility for all content in the submitted manuscript.

### 4.1. Sample Coding and Identification Data

Each sample was assigned an anonymized laboratory code from A to K. For two products, consecutive production batches were analyzed and coded E1–E3 and K1–K3. Thus, 11 commercial formulations were examined, and because three batches each were analyzed for two of them, the total number of analytical determinations (hereafter “analytical samples”) was 15 (11 − 2 + 6 = 15). This design allowed both a comparison between different products and an assessment of the consistency of results at the production-batch level. For each sample, the following were recorded from the packaging and purchase documentation: trade name, batch number, form of preparation, declared composition, minimum-durability or use-by date, source of purchase, condition of the packaging, and the presence or absence of any declaration regarding irradiation.

### 4.2. Thermoluminescence Analysis: Physical Basis and Procedure

All analyses were performed at the Independent Laboratory for the Identification of Irradiated Food at the Institute of Nuclear Chemistry and Technology (INCT, Warsaw, Poland), accredited by the Polish Centre for Accreditation to ISO/IEC 17025 [29]. The thermoluminescence method was applied in accordance with standard PN-EN 1788:2002 [28].

The method exploits the ability of naturally occurring silicate minerals (mainly quartz and feldspar), present as impurities in the plant matrix, to store the energy of ionizing radiation as electrons trapped at crystal-lattice defects. During controlled heating, the trapped electrons are released, return to lower-energy states, and emit light that is recorded by the TL reader [19,20,21]. A broad emission maximum in the stable high-temperature region—typically around 200–250 °C—is of particular interpretive significance, as it corresponds to the emptying of deeper, more stable electron traps and is considered a strong indication of prior irradiation of the mineral material [19,20,21].

The analytical procedure comprised the following steps:

1. Isolation of the mineral fraction. A 100–150 g portion of the sample was dissolved in water and sonicated for approximately 15 min. The beaker was then left to stand for 10–15 min to allow the mineral fraction to settle, after which the excess water was decanted. The remaining sediment was transferred to a centrifuge tube. Sodium polytungstate (SPT) solution, Na_6_[H_2_W_12_O_40_]·xH_2_O, at a density of 2 g cm^−3^, was used as the heavy liquid. To the mineral fraction at the bottom of the tube, 2–3 cm^3^ of the SPT solution (density 2 g cm^−3^) was added; the tube was shaken and then sonicated for approximately 3–5 min. The contents were centrifuged for 2 min at 1000× *g* to allow all mineral material to settle at the bottom of the tube. The isolated silicate minerals (approximately 1–2 mg) were deposited onto measurement cups and dried overnight in a laboratory oven at 50 °C ± 5 °C. The isolated minerals act as natural dosimeters.
2. Natural-signal measurement (TL1). The isolated minerals were deposited on stainless (acid-resistant) steel measurement cups (Risø-brand, approximately 10 mm in diameter, wall thickness approximately 0.25 mm) and heated in a Risø TL/OSL System reader, model TL-DA-20 (Risø National Laboratory, Roskilde, Denmark; last upgraded in 2024), up to a maximum temperature of 450 °C at a heating rate of 6 °C s^−1^. Glow curves were registered over the temperature range 70–450 °C (minimum detection level, MDL = 990 counts). The measurement chamber was continuously purged with high-purity nitrogen (grade 6.6) at a constant flow rate throughout the measurement.
3. Calibration measurement (TL2). Following the TL1 reading, the same mineral aliquot was irradiated with a reference dose of 1 kGy from a ^60^Co gamma source (Gamma Chamber 5000 (Board of Radiation and Isotope Technology [BRIT], Navi Mumbai, India), installed at the Centre for Radiation Research and Technology, INCT, in April 2010; nominal ^60^Co activity 13,892 Ci; dose rate 9.99 kGy h^−1^) and re-annealed overnight at 50 °C ± 5 °C. The sample was then re-measured (Glow 2) under conditions identical to those used for TL1: temperature range 70–450 °C, heating rate 6 °C s^−1^, MDL = 990. This second reading was used to determine the sensitivity of the mineral matrix and to correct for variable mineral recovery between samples.

Final classification was based not on a single numerical value alone, but on the overall evaluation of signal intensity and thermal stability, the position of the emission maximum, and the quality of the whole glow-curve profile.

### 4.3. Interpretation Criteria

The irradiation status of each sample was classified using the normalized TL ratio, defined as the ratio of the natural to the calibration luminescence intensity (TL1/TL2). In accordance with PN-EN 1788:2002 [28], the following criteria were adopted:

- Irradiated sample: TL1/TL2 > 0.1;
- Non-irradiated sample: TL1/TL2 ≤ 0.1.

An additional necessary condition confirming irradiation was the qualitative analysis of the glow curve: a sample was considered positive only if the TL emission maximum occurred in the stable high-temperature region (approximately 200–250 °C), which allows the irradiation signal to be distinguished from parasitic luminescence [23].

### 4.4. Quality Assurance and Quality Control (QA/QC)

Only results obtained in a laboratory accredited to ISO/IEC 17025 were considered. The assay was based on a validated reference method, and the reliability of results was supported by internal equipment-control procedures and by activities specific to laboratories operating within an accreditation system. This substantially reduces the risk of misclassification arising from improper sample preparation or equipment instability.

### 4.5. Label Audit

For each sample, the label was inspected for any declaration indicating irradiation (in particular the wording “irradiated” or “treated with ionizing radiation”), and the label information was compared with the TL results in accordance with the applicable EU and national provisions [12,13,14,15,16].

### 4.6. Statistical Analysis

To test the hypothesis of selective irradiation of the raw material, the samples were divided into two groups: preparations consisting of valerian root alone or valerian root with black pepper (*n* = 7), and the remaining supplements—multi-ingredient preparations containing valerian together with other components, and non-valerian comparators (*n* = 8). Between-group differences in the proportion of positive results were evaluated using Fisher’s exact test, with statistical significance set at *p* < 0.05. The corresponding proportions are reported in Section Results.

## 5. Conclusions

1. Thermoluminescence analysis revealed characteristics of prior irradiation in 46.7% of the analytical samples (representing 11 commercial dietary-supplement formulations available at retail).
2. All positive results concerned exclusively products containing valerian root, whether alone or with black pepper, indicating a strong association between the raw material and/or supply chain and a positive TL profile.
3. The repeatability of positive results across independent batches of two products suggests that the phenomenon is systematic rather than incidental.
4. No positive sample carried a label declaration regarding irradiation or exposure to ionizing radiation, indicating a significant discrepancy between the analytical result and the information provided to the consumer.
5. The results may serve as a premise for considering potential technological food adulteration; however, confirming this interpretation would require additional data from manufacturer and supply-chain documentation.
6. Thermoluminescence analysis confirmed its usefulness as a tool for independent verification of the technological authenticity of plant-based dietary supplements.
7. Further studies should include a larger number of products, a wider range of plant raw materials, more complete documentation of raw-material origin, and assessment of the effects of irradiation on the phytochemical profile and functional quality of valerian-root products.

## Figures and Tables

**Table 1:** Results of thermoluminescence analysis and label audit for the examined dietary supplements. No label declaration concerning irradiation was present in any of the 15 samples.

Sample Code	Main Declared Ingredients	Form	TL Result	Interpretation
A	Valerian root (*Valeriana officinalis*)	Capsules	Positive	Irradiated
B	Bacopa (*Bacopa monnieri*)	Capsules	Negative	Non-irradiated
C	Turmeric + vitamin E	Capsules	Negative	Non-irradiated
D	Garcinia cambogia	Capsules	Negative	Non-irradiated
E1	Valerian root + black pepper	Capsules	Positive	Irradiated
F	Saffron, valerian, GABA, magnolia, melatonin, lemon balm, tryptophan, Asian pennywort	Tablets	Negative	Non-irradiated
G	Niacin, riboflavin, vitamin B6	Capsules	Negative	Non-irradiated
H	Valerian	Capsules	Negative	Non-irradiated
I	Hops, fennel, valerian, lemon balm	Tablets	Negative	Non-irradiated
J	Hops, ashwagandha, valerian, melatonin, magnesium	Tablets	Negative	Non-irradiated
K1	Valerian root	Capsules	Positive	Irradiated
K2	Valerian root (batch 2)	Capsules	Positive	Irradiated
K3	Valerian root (batch 3)	Capsules	Positive	Irradiated
E2	Valerian root + black pepper (batch 2)	Capsules	Positive	Irradiated
E3	Valerian root + black pepper (batch 3)	Capsules	Positive	Irradiated

**Table 2:** Summary of results by product type.

Product Group	No. of Analytical Samples	Positive	Negative	Note
Valerian root as main ingredient	4 (A, K1–K3)	4	0	All samples positive
Valerian root + black pepper	3 (E1–E3)	3	0	All samples positive
Multi-ingredient products with valerian	4 (F, H, I, J)	0	4	No signs of prior irradiation
Non-valerian comparators	4 (B, C, D, G)	0	4	No signs of prior irradiation
Total	15	7	8	Positive results exclusively in simple valerian preparations

## Data Availability

Detailed data are available from the authors.

## Abbreviations

The following abbreviations are used in this manuscript:

INCT	Institute of Nuclear Chemistry and Technology
PN-EN	Polish Standard (European Norm)
EU	European Union
SPT	Sodium Polytungstate
TL	Thermoluminescence
